# Supplementary material for: Relationship between Circulating Lipids and Cytokines in Metastatic Castration-Resistant Prostate Cancer
Source: Cancers (Basel). 2021 Oct 1;13(19):4964. doi: 10.3390/cancers13194964 (PMC8508038; doi:10.3390/cancers13194964)
Supplement: Supplementary file 1 [file cancers-13-04964-s001.zip › Supplementary Information_figures tables.pdf]

Table of contents.

Table of contents. 1
Figure S1. 2
Figure S2. 3
Figure S3. 4
Figure S4. 5
Figure S5. 6
Table S1. 7
Table S2. 7

Figure S1.

(a) Kaplan-Meier curves of the association of the three-lipid signature (3LS) at baseline with PSA-PFS in Cohort 1 and rPFS in Cohort 2a; (b) Forestplot of hazard ratios of rPFS for baseline lipid levels in Cohort 2a; (c) Forestplot of hazard ratios of rPFS for baseline cytokine levels in Cohort 2a.

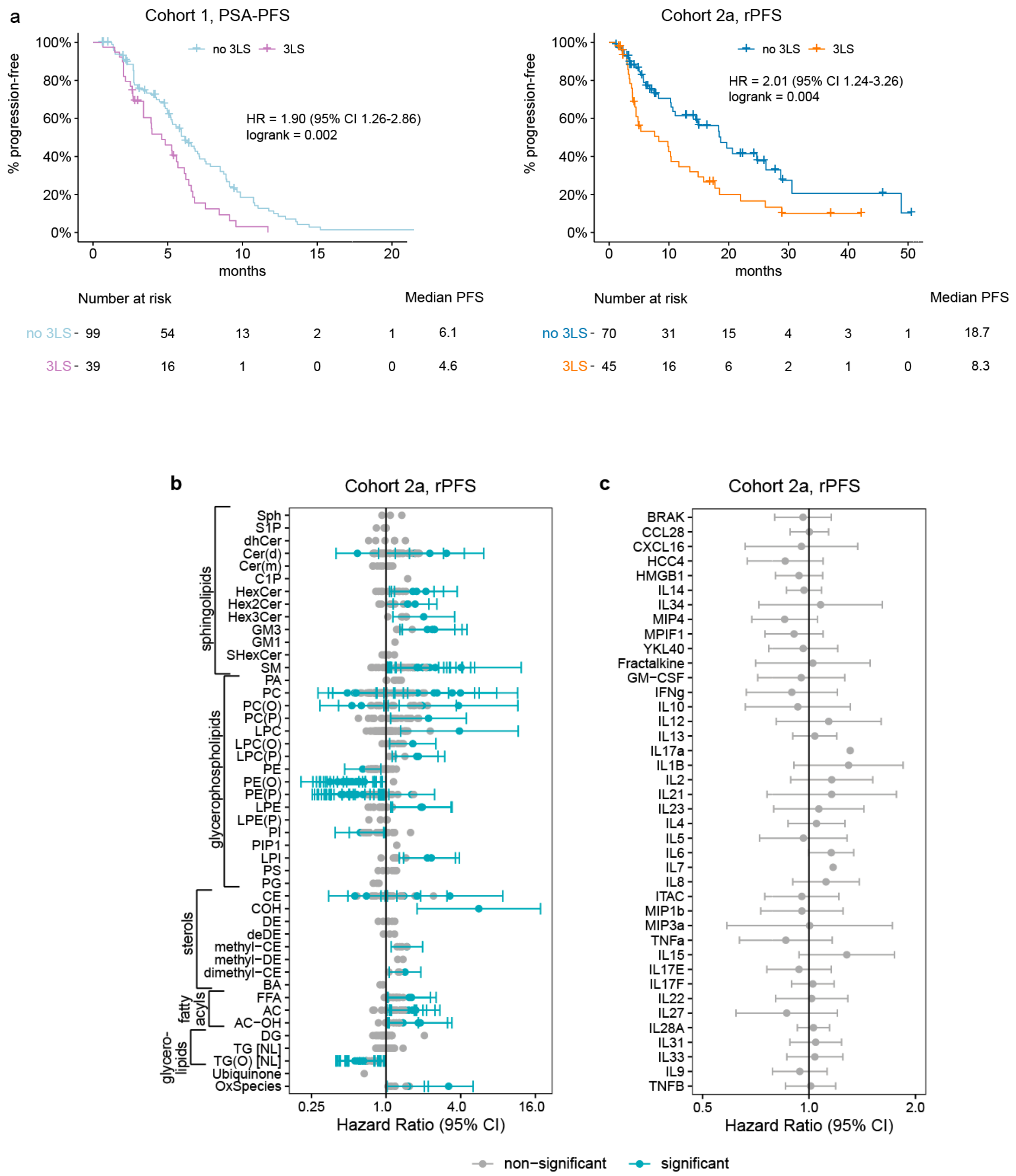

Figure S2.

Heatmaps of Pearson coefficient of correlation and corresponding p-value for the correlation between blood neutrophil to lymphocyte ratio and baseline levels of 121 prognostic lipids or cytokines in Cohort 2a.

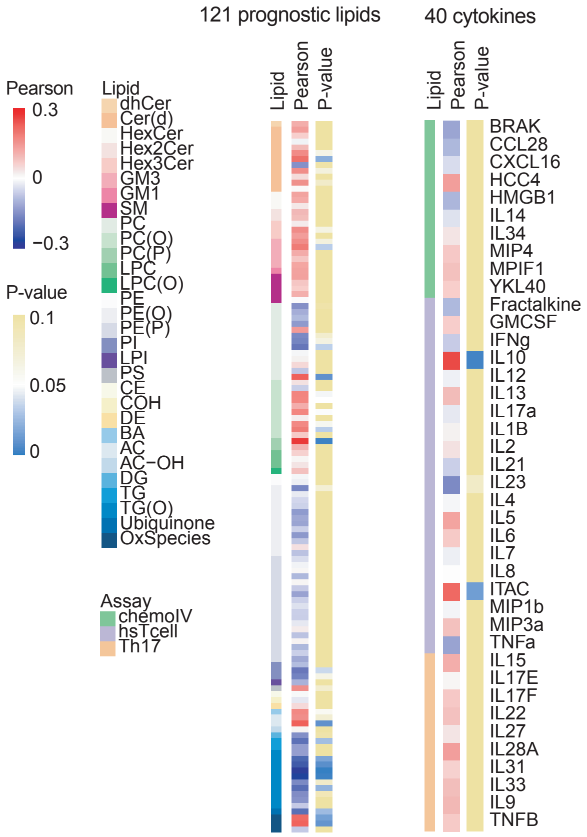

Figure S3.

PCA of end of treatment (EOT) plasma samples for Cohort 2b according to their lipid profiles (121 prognostic lipids) or cytokine profiles (40 cytokines).

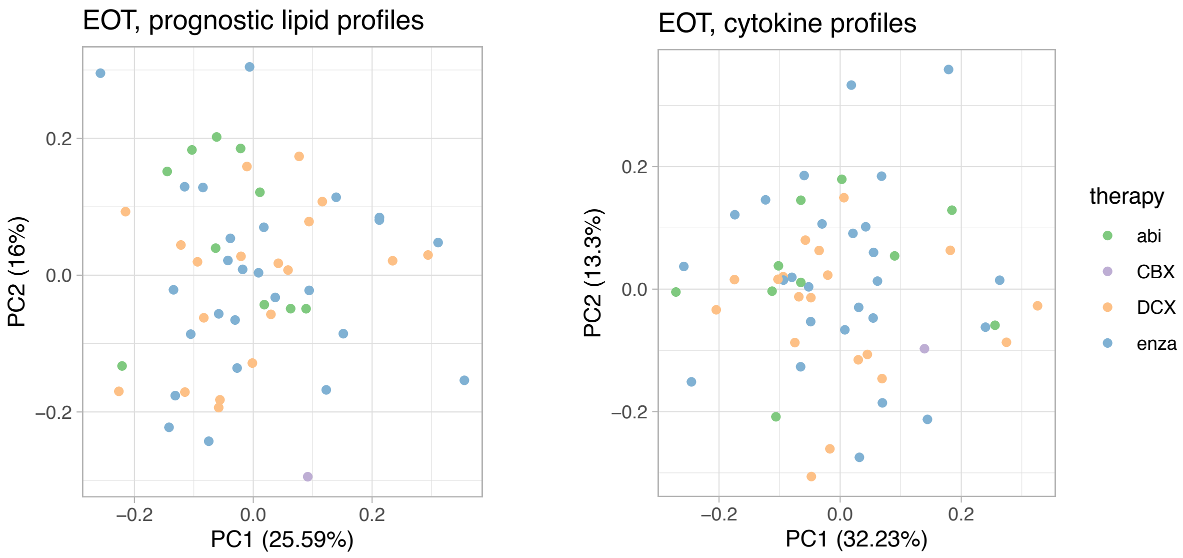

Figure S4.

Hierachical clustering of 56 pairs of matched baseline and EOT plasma samples for Cohort 2b, with heatmaps of the lipid or cytokine levels. Samples are clustered according to the profiles of 121 prognostic lipids (a), or 40 cytokines (b). Matched baseline and EOT samples that paired together (cluster next to each other) are highlighted in orange.

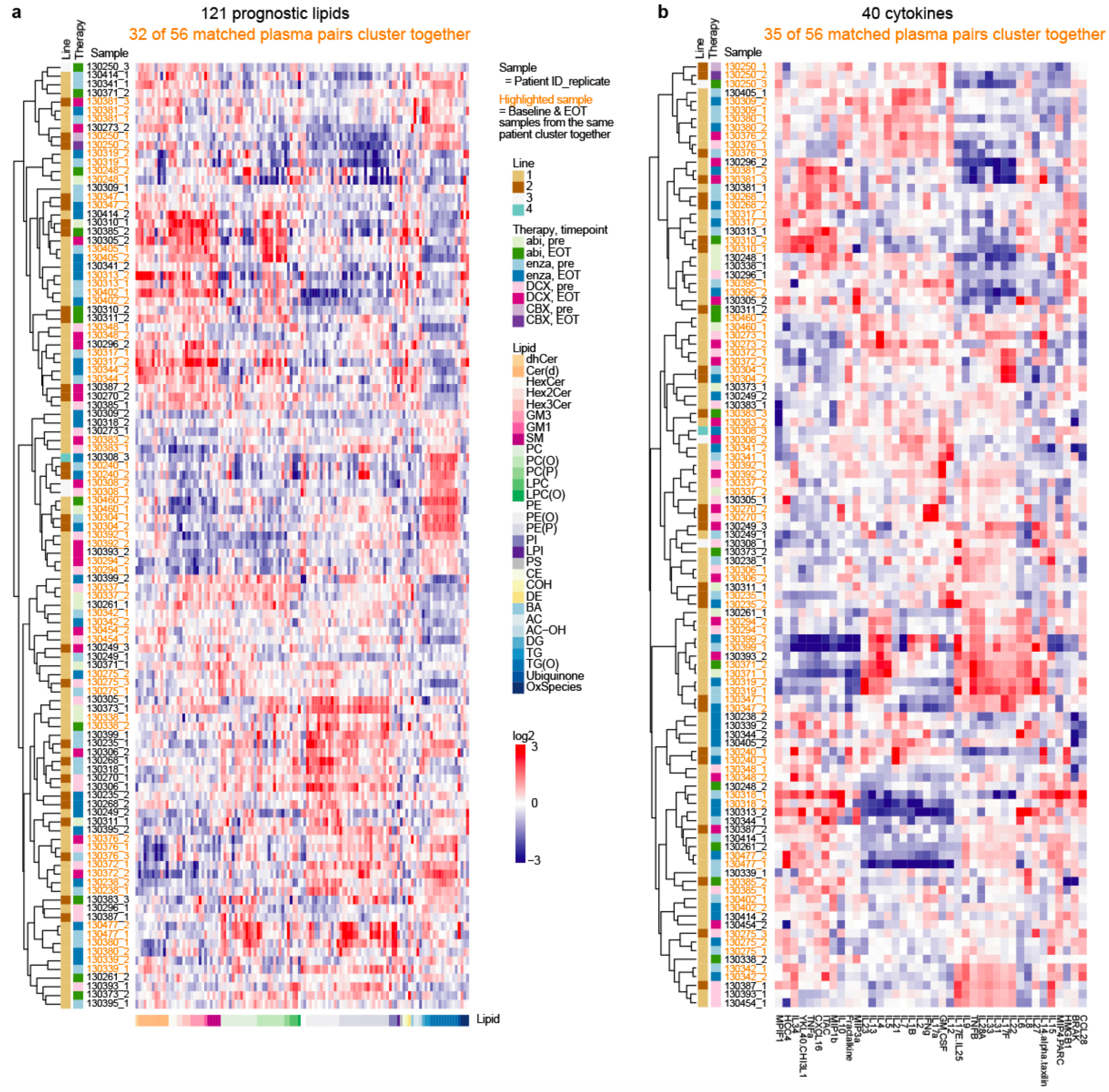

Figure S5.

Plasma levels of sphingosine-1-phosphate (S1P) are correlated to the plasma levels of prognostic ceramides: (a) Scatterplots of log2 pmol/ml levels of the predominant forms of S1P (S1P(d18:1), S1P(d18:2)) versus prognostic ceramides with the corresponding sphingoid base (d18:1 or d18:2) in Cohort 2a; (b) Ceramide structure and nomenclature defining the sphingoid base; (c) Metabolism of ceramide into S1P; (d) Boxplots of plasma levels of S1P in Cohort 2a showing that the d18:1 and d18:2 are the major S1P species in plasma.

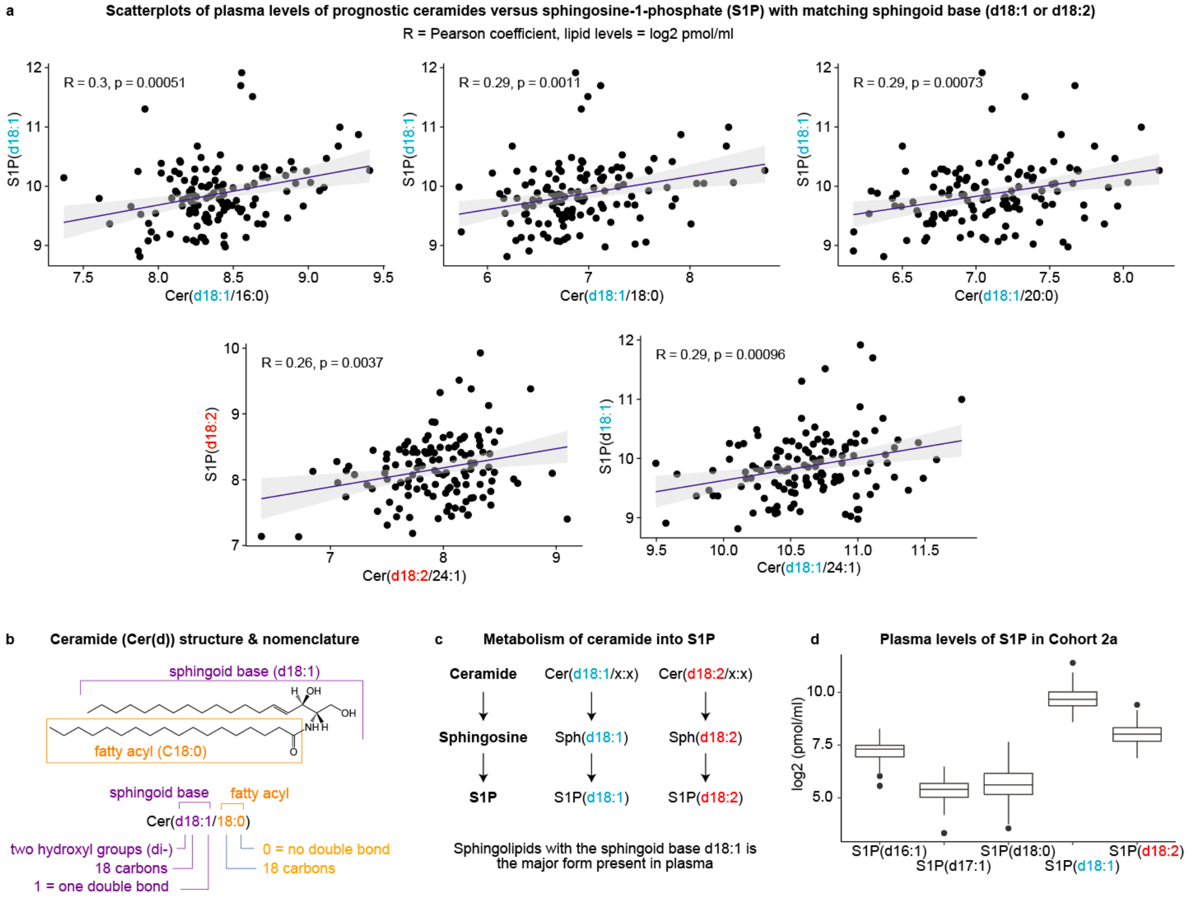

Table S1.

Significantly enriched lipid types among circulating lipids associated with overall survival in Cohort 1.

Association with overall survival	Lipid type	Number of prognostic lipid species	Total lipid species in dataset	Fisher's exact p-value
Higher levels = shorter overall survival	SM	10	20	0.0001
	GM3	5	6	0.0003
	Cer(d)	4	6	0.005
	Hex3Cer	3	6	0.04
	PC(P)	4	10	0.04
Lower levels = shorter survival	TG	14	41	0.0002
	LPC	6	18	0.02

Table S2.

Significantly enriched lipid types among circulating lipids associated with overall survival in Cohort 2b.

Association with overall survival	Lipid type	Number of prognostic lipid species	Total lipid species in dataset	Fisher's exact p-value
Higher levels = shorter overall survival	GM3	5	6	0.000007
	Cer(d)	10	49	0.0009
	PC(O)	6	22	0.002
	Hex3Cer	3	6	0.005
	OxSpecies	3	9	0.02
Lower levels = shorter overall survival	PE(O)	12	14	$1x10^{-11}$
	PE(P)	18	53	0.0000002
	TG(O)	10	20	0.000002
	PC	11	68	0.04
